# Recommended nitrogen fertilization enhances soil carbon sequestration in China’s monsoonal temperate zone

**DOI:** 10.7717/peerj.5983

**Published:** 2018-11-16

**Authors:** Shaofei Jin

**Affiliations:** Department of Geography, Ocean College, Minjiang University, Fuzhou, China; Key Laboratory of Regional Climate-Environment for Temperate East Asia (RCE-TEA), Institute of Atmospheric Physics, Chinese Academy of Sciences, Beijing, China; Fujian Provincial Key Laboratory of Information Processing and Intelligent Control (Minjiang University), Fuzhou, China

**Keywords:** Root biomass distribution, Carbonate, Winter wheat, Soil organic carbon

## Abstract

China consumes more than one-third of the world’s nitrogen (N) fertilizer, and an increasing amount of N fertilizer has been applied over the past decades. Although N fertilization can increase the carbon sequestration potentials of cropland in China, the quantitative effects of different N fertilizer application levels on soil carbon changes have not been evaluated. Therefore, a 12-year cultivation experiment was conducted under three N fertilizer application levels (no N fertilizer input, the recommended N fertilizer input after soil testing, and the estimated additional fertilizer input) to estimate the effect of N addition on soil carbon changes in the root layer (0–80 cm) and non-root layer (80–200 cm) using a within-study meta-analysis method. The results showed significant declines in the soil inorganic carbon (SIC) in the root layers and significant growth in the SIC in the non-root layers under N fertilizer input. The soil organic carbon (SOC) in the root layers and the non-root layer significantly decreased under all the treatments. In addition, the recommended N fertilizer application level significantly increased the SOC and soil total carbon stocks compared with the future N fertilizer application level and no N input, while the future N fertilization significantly decreased the SIC and soil total carbon compared with no N input. The results suggest that N fertilization can rearrange the soil carbon distribution over the entire soil profile, and the recommended N fertilization rather than excess N input can increase the soil carbon stock, which suggests that the national soil testing program in China can improve the soil carbon sequestration potential.

## Introduction

The amount of nitrogen (N) fertilizer applications has increased rapidly since the 1980s (Lassaletta et al., 2014; Lu & Tian, 2017; Prud’homme, 2005). Although excessive N input has induced negative ecological and environmental impacts (Liu et al., 2013), such as soil acidification (Guo et al., 2010) and eutrophication (Conley et al., 2009), N fertilizer plays a significant role in food security (Spiertz, 2010; Tilman et al., 2011), e.g., by eliminating hunger (Borlaug, 1971) and improving cereal production (Tilman et al., 2002). In addition, N inputs can alter the soil carbon stocks when accompanied by different cultivation managements (Halvorson, Reule & Follett, 1999; Khan et al., 2007; Neff et al., 2002). For instance, the soil carbon stocks benefit from the application of manure and N fertilizer in four long-term experiments in Germany and China (Ludwig et al., 2011).

Studies on the impacts of excess N fertilizer application to the carbon cycle have been widely concentrated on the soil organic carbon (SOC) dynamics (Alvarez, 2005; Khan et al., 2007). However, the soil carbon includes both a SOC pool and a soil inorganic carbon (SIC) pool in the arid/semiarid ecosystems (Lal, 2002; Lal, 2004a). Most studies have overlooked the impacts of the N inputs on the SIC (Monger et al., 2015; Schlesinger, 1982). The global SIC contributes to approximately 30% of the world’s total carbon in the upper 1 m depth of soil (Batjes, 1996; Lal, 2004b), occupies more carbon than the SOC in the dryland ecosystem (Lal, 2004a), and stores more carbon in the deep soil (Díaz-Hernández, 2010). Using China as an example, the SIC stores quantities comparable to the amount of SOC in China (Li et al., 2007). Specifically, the SIC was estimated to be 55.3 PgC (1Pg C = 10^15^ g C) (Wu et al., 2009), and the SOC in China was estimated to be 70.3 PgC (Wu, Guo & Peng, 2003a). In addition, agricultural practices could alter the allocation of soil carbon (Guo & Gifford, 2002; Sun et al., 2010; Wu, Guo & Peng, 2003b). Modern agricultural practices, primarily the N input (Guo et al., 2010), have increased the SOC by 0.437 PgC (Sun et al., 2010) and decreased the SIC by 1.6 PgC (Wu et al., 2009) in China’s agricultural soil over the past three decades. In addition, a series of studies conducted in arid and semiarid farms showed that the SIC had a greater accumulation rate than the SOC under four long-term N fertilizer experiments (Wang et al., 2015; Wang et al., 2014b; Zhang et al., 2015). However, the SIC in deep soil (below 100 cm) may be lost due to soil acidification caused by the long-term N fertilization (Jin, Tian & Wang, 2018). Considering the large stocks of the SIC in the deep soil, small changes in the SIC in the deep soil carbon may change the soil carbon stocks. Despite its importance, how the soil carbon in the deep soil changes under long-term N fertilization experiments is still unclear.

China consumes more than 30% of the total fertilizer in the world and has continuously applied more N fertilizer since the 1980s (Ju et al., 2004; Zhu & Chen, 2002). This increased fertilizer usage has resulted in the miracle of utilizing 7% of the world’s croplands to feed 22% of the world’s population (Gong, 2011). In addition, the government noted the importance of using sustainable agricultural developments at the beginning of the 21st century. The key phase of China’s No 1 Central Document for 2005 was to improve the capacity of agricultural production through soil testing and fertilizer recommendations. Although an N fertilizer application rate was recommended, farmers still applied more N fertilizer than necessary (Ju et al., 2004). Little is known about how the recommended N fertilizer application rate and the actual N fertilizer application by farmers affect the soil carbon changes. Therefore, explicitly estimating the effect of soil testing and fertilizer recommendation in China will aid in determining the changes in the carbon stock for the crop soil in the country.

In this study, a long-term N fertilization experiment was carried out by setting three N fertilizer application levels (no N fertilizer input, the recommended N fertilizer input after soil testing, and the estimated further fertilizer input of farmers) beginning in 2002 in Yangling. The experimental site is located in China’s monsoonal temperate zone. This zone occupies 49.4% of the total cropland areas, 49.2% of the total crop yields, 33.7% of the total SOC stock (Wu, Guo & Peng, 2003b), and 24.8% of the total SIC stock (Wu et al., 2009) of China. In this study, I use a within-study meta-analysis to evaluate the changes in the soil carbon stocks (up to a depth of 200 cm) under the recommended and projected N application rates. Considering that the SIC in deep soil (below 100 cm) can be loss due to soil acidification induced by the long-term N fertilization (Jin, Tian & Wang, 2018), and the large stocks of the SIC in the deep soil (Díaz-Hernández, 2010), I hypothesized that the soil carbon in the deep soil will respond to the N fertilization after more than one decade’s cultivation (Hypothesis 1). In addition, increasing the soil carbon sequestration potential contributes to the sustainable agricultural developments (Lal, 2004b; Pretty, 2008), I hypothesized that if the program of soil testing and nitrogen recommendation succeeds, the recommended N fertilizer application levels will enhance the soil carbon stocks (Hypothesis 2).

## Materials & Methods

### Site description and experimental design

Using a monoculture of winter wheat (*Triticum aestivum*), a long-term agricultural experiment to investigate the effects of nitrogen applications on agroecological soil dynamics was initiated in October 2002 at the Long-term Experimental Farm (34^∘^17′44″N, 108^∘^04′10″E) of the Northwest Agriculture and Forest University in the Yangling District, Shannxi Province, China. This experimental farm is located in the center of China with an altitude of 525 m above sea level. The mean annual temperature is 13 °C, and the mean annual precipitation is 600 mm with 60% of the rainfall occurring between July and September. The soil is classified as an Earth-cumuli-Orthic Anthrosol according to the Chinese Soil Taxonomy (Shi et al., 2006) and a Udic Haplustalf according to the USDA system (Staff Soil Survey, 1999; Wang et al., 2014a). The parent material of the soil is loess. The original selected soil properties for the first 20 cm depth (Wang et al., 2014a were as follows: pH of 8.25) SOC of 8.00 g/kg, SIC of 10.67 g/kg, NO_3_–N of 5.43 mg/kg, NH_4_–N of 2.41 mg/kg, and a bulk density of 1.21 g/cm^3^. The soil texture is loam comprising 33.3% sand, 41.7% silt, and 25.0% clay.

Nine plots with areas of 59.4 m^2^ (6 m by 9.9 m) were designated in this experiment. Three N (urea, N = 46%) application rates of 0 kg/ha (N0), 120 kg/ha (N120), and 240 kg/ha (N240) with three replicates were randomly and evenly applied to the nine plots. The N0, N120, and N240 represent the control treatment (without N input), the recommended N application rate after soil testing (the optimal N input), and the doubled N application rate (the future N input), respectively. In addition, all the other operations during the growing season remained consistent with the local farming methods. In short, phosphate fertilizer (superphosphate, P_2_O_5_>16%) was applied at a rate of 100 kg P_2_O_5_/ha in each plot as the base fertilizer, and the winter wheat was sown with 150 kg/ha in 30 rows in early October and harvested in the middle of June the following year.

### Soil samples and analyses

Soil samples in each plot were collected from 10 soil layers distributed evenly between the depths of 0 −200 cm using a soil auger (4 cm diameter). Three cores were collected randomly from each plot. The samples from the same depth in each plot were thoroughly mixed and the roots air-dried, and other visually identified organisms, such as residual roots, were removed. The air-dried samples were ground to fully pass through a 2-mm sieve. They were divided into two parts: one was used to measure the soil carbon after thoroughly passing through a 0.15 mm sieve, and the other was stored for validation. The soil carbon was measured using a multi N/C 2100 (Analytic JENA, AG-Germany) at the Institute of East China Sea Fisheries Research, Chinese Academy of Sciences. The soil total carbon (STC) was measured using the direct combustion of 10–20 mg of soil. The SOC was measured by pretreating 10−20 mg of the soil with 1 M HCl for 24 h to remove the carbonate and processing the sample using the same procedure as the STC. The SIC was calculated as the difference between the STC and SOC. The soil bulk density was determined using the oven-drying method to measure the soil mass and core volume (Soil Survey Staff, 2011).

### Modeling the distribution of the root mass of winter wheat

**Figure 1:**
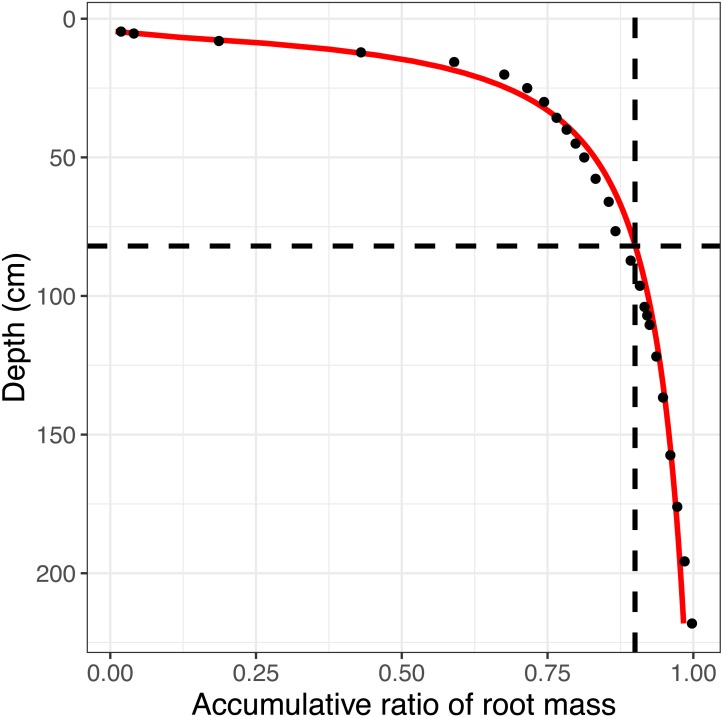
The simulated accumulative ratio of the root biomass. The red line is the modeled distribution, and the dots are the observations. The dashed lines show the soil depth (80 cm) with 90% of the root biomass.

In this study, the root mass distribution (RMD) was used to divide the soil depth into two layers: the root layer with 90% of the root mass and the non-root (subsoil) layer with 10% of the root mass. As reported in a previous study (Zhang, Pei & Chen, 2004), which measured the RMD of winter wheat in different growth stages, the root biomass within the depth of 80 cm contributed 94.1% of the total root biomass during the first 190 days after sowing and 87.7% in the early milk stage (220 days after sowing). In addition, a vertical distribution of RMD was observed and an equation was proposed (Liu et al., 2008). I simulated the accumulative ratio of the root mass along the soil profile (Fig. 1). The simulation showed that the 80 cm soil depth is an acceptable depth to use to divide the two layers in this study.

### Statistical analysis

#### Within-study meta-analysis method

Due to the 12-year period of this study, several uncontrolled factors (e.g., seed sowing/yield by different people) in the field experiments may affect the soil carbon, although the study only attempted to vary three N application rates. Therefore, a within-study meta-analysis method rather than a single analysis of variance (ANOVA) was applied to investigate the relationship between the soil carbon changes and N fertilization. A within-study meta-analysis (Van Zandt, 2007) is useful to combine the results from several independent results obtained in a systemic study when accounting for uncontrolled variation between experiments. The advantages of a within-study meta-analysis include (1) establishing a statistical significance with studies that have conflicting results, (2) developing a more correct estimate of the effect magnitude, (3) providing a more complex analysis of risks and benefits, and 4) examining subgroups with individual studies. Therefore, typical ANOVA results, e.g., the *F* value, and a post hoc comparison, e.g., LSD, were not shown in this study. The results of the within-study meta-analysis are illustrated naturally but show a 95% confidence interval (CI) of the effect size. In this study, if the 95% CI of the effect size overlapped with zero, significant changes will be detected. The raw data and R code are provided in the supplementary files 1 and 2, respectively.

The original soil carbon concentrations at the depths of 0−200 cm in 2002 were extracted from previous studies (Dong, Cai & Zhou, 2013). The experimental soil carbon was measured from this study. For each soil layer, an effect size (response ratio) was used to express the magnitude of the impact on the soil carbon (SOC, SIC, and STC) under different N application rates after 12 years of cultivation. The effect size was calculated as the log ratio of the mean soil carbon in 2014 and the mean in 2002: effect size = log( *SC*_2014_*/SC*_2002_). The corresponding sample variance (Gurevitch, Curtis & Jones, 2001; Osenberg, Sarnelle & Goldberg, 1999) was calculated as follows,

(1) \begin{eqnarray*}{V}_{ES}= \frac{SD(S{C}_{2014})^{2}}{{N}_{2014}(S{C}_{2014})^{2}} + \frac{SD(S{C}_{2002})^{2}}{{N}_{2002}(S{C}_{2002})^{2}} \end{eqnarray*}

where *SC*_2014_ and *SC*_2002_ are the soil carbon content at the end and beginning of the experiment, respectively; *N*_2014_ and *N*_2002_ are the sample numbers at the end and beginning of the experiment, respectively, and SD(*SC*_2014_) and SD (*SC*_2002_) are the standard variances of the soil carbon at the end and beginning of the experiment, respectively. The effect sizes of the different soil layers were pooled weighted by soil bulk density by the root layer, the non-root layer, and the whole soil layer (0–200 cm) for use within the study meta-analysis. The pooling effect size was obtained by conducting a separate meta-analysis for the dataset at each layer. No significant differences between the soil carbon in 2002 and 2014 were found when the 95% CI overlapped with 0. All these analyses were implemented using the *metafor* package of *R* (R Core Team, 2018; Viechtbauer, 2010).

#### Stocks of soil carbon

The stocks of soil carbon (SOC, SIC, and STC) were calculated (Batjes, 1996; Wang et al., 2010) as follows:

(2) \begin{eqnarray*}{C}_{stocks}=\sum _{i=1}^{n}{C}_{i}\ast B{D}_{i}\ast {H}_{i}\ast (1-{S}_{i})\end{eqnarray*}

where *C*_stocks_ is the carbon stocks (kg/m^2^) of the SOC, SIC, and STC in the root layer (0∼80 cm), non-root layer (80∼200 cm), and whole soil profile (0∼200 cm), and *C*_*i*_, *BD*_*i*_, *H*_*i*_, and *S*_*i*_ are the soil carbon (gC/kg), bulk density (g/cm^3^), the soil depth (m), and the volume fraction of fragments >2 mm in the soil layer *i*, respectively. Due to the loess parents, the coarse fractions (>2 mm) was identified as zero.

#### Soil carbon stock changes between different nitrogen fertilization levels

The changes in the soil carbon between no N input, the recommended N fertilizer application rate, and the future N fertilizer application rate in China’s monsoonal temperate zone were calculated as follows:

(3) \begin{eqnarray*}{\mathrm{Changes}}_{i-j}={\mathrm{Carbon}}_{i}-{\mathrm{Carbon}}_{j}\end{eqnarray*}

(4) \begin{eqnarray*}{\mathrm{V ar}}_{i-j}= \frac{{S}_{i,N0}^{2}}{{N}_{i}} + \frac{{S}_{i,j}^{2}}{{N}_{i}} \end{eqnarray*}

where *i,j* represents the different N fertilizer application level (N0, N120 and N240); Var represents the estimated variance of the changes in soil carbon, and *S* represents the standard deviation of the corresponding carbon.

## Results

**Figure 2:**
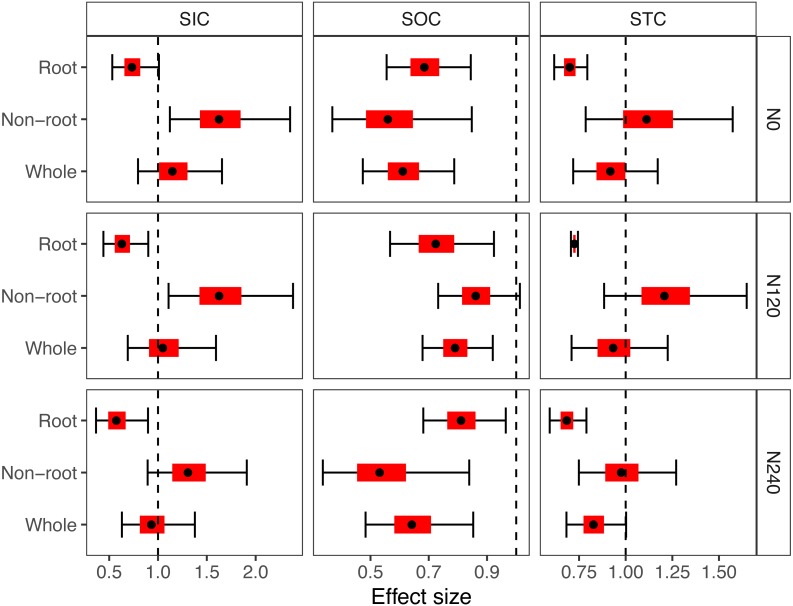
Effect of the sizes of the soil carbon after 12 years of cultivation under three N application levels in the root, non-root, and the whole layer at a 0–200 cm soil depth. The solid points represent the mean effect size for each layer. The horizontal black error bar and the red bars represent the 95% confidence interval (CI) and the 50% CI of the effect size, respectively. The vertical dashed lines represent the reference lines. There was no significant difference between the soil carbon in 2002 and 2014 when the 95% CI overlapped the dashed lines. Root: root layer, 0–80 cm; non-root: 80–200 cm, whole: 0–200 cm.

The changes in the soil carbon in the different soil layers after 12 years of cultivation under different N application rates are shown in Fig. 2. Significant declines in the SIC were found in the root layers under the N120 and N240 treatments, while a significant increase in the SIC was found in the non-root layers under the N0 and N120 treatments. The SOC in 2014 had significantly decreased relative to the SOC in 2002 (none of the 95% CIs overlapped with the reference line). In addition, the pooled effects of the different responses of the SOC, SIC, and STC to the N fertilizer application at the root layer declined significantly, while the STC in the whole soil profile showed no significant changes after the long-term experiment. Specifically, after the 12-year cultivation, the N0, N120, and N240 treatments caused changes in the STC of −8.24% (95% CI [−28.15–17.18]), −6.73% (95% CI [−29.01–22.54]), and −17.30% (95% CI [−31.75–0.22]), respectively.

**Figure 3:**
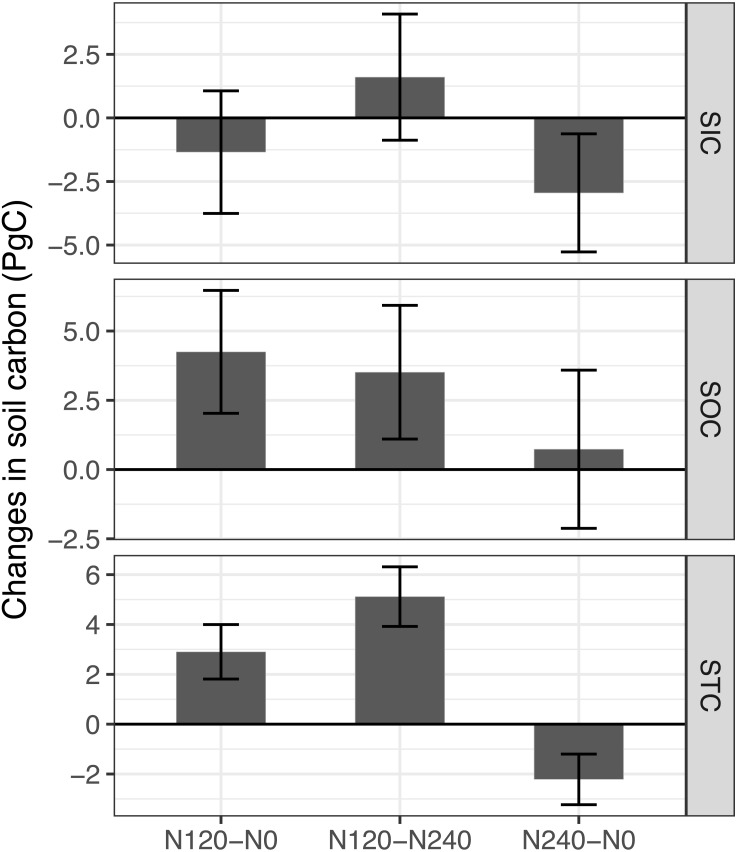
Estimated contributions of different nitrogen fertilizer application levels to the carbon changes relative to the changes of no nitrogen input in the monsoonal temperate zone in China. The error bars represent the 95% confidence interval (95% CI). N120-N0, N240-N0, and N240-N120 represent the soil carbon changes between the recommended N fertilization and no N input, future N fertilization and no N input, future N fertilization and recommended N fertilization, respectively. Significant changes will be found when the 95% CI does not overlap with zero.

Figure 3 shows the estimated soil carbon stock changes between no N input, the recommended N fertilizer application rate, and the future N fertilizer application rate in China’s monsoonal temperate zone. The recommended N fertilizer application level significantly increased the SOC and STC stocks compared with the future N fertilizer application level and no N input (Fig. 3). Specifically, for the SOC, the recommended N application level will cause an increase of 4.25 (95% CI [2.03–4.67]) PgC and 3.51 (95% CI [1.10–5.93]) PgC compared with no N input and the future N fertilization level, respectively. For the STC, the recommended N application level will cause an increase of 2.90 (95% CI [1.81–4.00]) PgC and 5.11 (95% CI [3.92–6.31]) PgC compared with no N input and the future N fertilization level, respectively. In addition, the future N fertilization can significantly decrease the SIC (−2.95, 95% CI [−5.27, −0.63] PgC) and STC (−2.21, 95% CI [−3.23, −1.20] PgC) compared with no N input.

## Discussion

I evaluated the effect of different N fertilizer application levels, representing optimal and projected levels, on the distribution of soil carbon and on the soil carbon stocks. The results supported the two hypotheses proposed: Hypothesis Hypothesis 1, which will illustrate the responses of the deep soil carbon to the N fertilization, and Hypothesis 2, which will reflect the role of the N fertilization recommendation in the carbon sink potential.

### Effects of nitrogen fertilization levels on the soil carbon stocks

The conventional agricultural soil sample depths are 40 cm for Regosols and 100 cm for Fluvisols because of the rooting depth (Díaz-Hernández, 2010). The sample depth in 90% of the studies estimating the soil organic carbon changes due to land use was less than 30 cm (Post & Kwon, 2000; Richter & Billings, 2015). In this study, a depth of 80 cm was considered to be the root layer, and a depth of down to 200 cm was considered to be the subsoil layer based on the simulated distribution of the root mass. After a long-term experiment, the SIC increased in the non-root layer and decreased in the root layer, while the SOC declined over the whole soil profile (Fig. 2). These results implied different responses of the soil carbon to N fertilization. Although numerous studies have investigated the effects of N fertilization on the SOC in the root layer (Jin, Tian & Wang, 2018; Khan et al., 2007), there was no agreement on the effects. The results were consistent with the data collected in the previous study (Khan et al., 2007). Although the SIC was recognized to be relatively stable (Schlesinger, 1985), the significant decline in the SIC in the root layer may be caused by the N input, which can induce soil acidification (Bolan, Adriano & Curtin, 2003). This acidification can subsequently result in the loss of the SIC in the root layer. In the subsoil layer, I predicted that the reprecipitation of calcium carbonate was the primary explanation for the increase in the SIC (Monger et al., 2015). This suggests that deep soil responds to agricultural management. Therefore, I believe that N fertilization can rearrange the overall soil carbon distribution.

To quantify the contribution of the different N fertilization application to the carbon stocks, I estimated the changes in the soil carbon under different N application rates. In this study, for the monsoonal temperate zone of China, I estimated that the recommended N application level can cause a 4.25 PgC increase in the SOC, while the future N application level did not significantly change the SOC (Fig. 3). Due to the soil acidification caused by the excess N input, the future N fertilization can significantly decrease the SIC by 2.95 PgC and the STC by 2.21 PgC. This result implied that the SIC should be considered to determine the overall responses of the soil carbon to N fertilization. Previous studies have shown that modern agricultural practices have increased the SOC by 0.437 Pg C (Sun et al., 2010), and human agricultural activity has caused a loss of 1.6 PgC of the SIC (Wu et al., 2009). In addition, land use changes have been estimated to have induced a loss of 7.1 PgC of the SOC in China (Wu, Guo & Peng, 2003b). My results showed that a suitable N fertilization level could increase the SOC more than the excessive N input. This finding implies that more SOC can be stored when the optimal N application is applied, while the soil carbon (SIC and STC) will decrease more with increasing N input. In addition, this study suggests that the national soil testing program in China will help improve the soil carbon sequestration potential.

### Policy implications

To achieve sustainable agricultural development, decreasing the N application and improving the N use efficiency is necessary for future agricultural practices (Ju et al., 2009). My results proved that the recommended N fertilizer application level can significantly enhance the SOC and STC, suggesting an improved carbon sink potential for the soil in China. These results could reflect the success of the program of the National N Recommendation of China, conducted since 2005, in the role of improving the potential in the soil carbon sink. However, farmers have continued to apply excess N over the past years (Ju et al., 2004). A regional questionnaire showed that the recommended N application rate is 123 kg/ha for winter wheat, while the actual mean N use by the farmers in this region is 178 kg/ha (Tong et al., 2004). Therefore, based on these results, more efforts are required to guide farmers to use the optimal N fertilization level.

### Limitations

Although the data supported the two hypotheses, two limitations can be identified based on the two hypotheses. To test Hypothesis 1, the carbon changes were estimated using the estimated carbon stocks in previous studies, all of which lack soil carbon data from the deeper layers. To overcome this, a more accurate benchmark of carbon stock, including estimates of deep soil stocks, is still needed. To test Hypothesis 2, due to the limited input, only the data from one long-term experiment were used to extrapolate the results to a regional scale, although I remained within a similar climatic zone. To overcome this limitation, combining more long-term experimental results will help to evaluate the effects of the recommended N fertilization at a regional scale.

## Conclusions

This study shows that the recommended nitrogen fertilization enhances soil carbon sequestration in China’s monsoonal temperate zone. In addition, the N fertilizer application can rearrange the soil carbon distribution over the entire profile, and more SOC can be stored when the optimal N fertilizer is applied, not when the N input is increased. In the future, more long-term experimental results are needed to combine to better evaluate the contribution of N fertilization to the soil carbon at a regional scale.

##  Supplemental Information

10.7717/peerj.5983/supp-1 File S1 Raw data

10.7717/peerj.5983/supp-2 File S2 R Code to replicate the analysis

10.7717/peerj.5983/supp-3 Supplemental Information 1 Observed data of the accumulative ratio of the root biomass at at a 0–200 cm soil depth in the Fig. 1

10.7717/peerj.5983/supp-4 Supplemental Information 2 Dataset for generating the effect sizes of soil carbon after 12 years of cultivation under three N application levels in the Fig. 2

10.7717/peerj.5983/supp-5 Supplemental Information 3 Dataset for generating the estimated contribution of different nitrogen fertilizer application levels to the carbon changes in the monsoonal temperate zone in China in the Fig. 3

## Additional Information and Declarations

### Data Availability

The following information was supplied regarding data availability: The raw data and R code are provided in the Supplemental Files.
